# Life Course Timing of Mortality Exposure and Fertility Behavior

**DOI:** 10.1007/s11113-025-09950-6

**Published:** 2025-04-30

**Authors:** Lauren Newmyer, Lisa McAllister, Nurul Alam, Mary K. Shenk

**Affiliations:** 1 Department of Sociology, Bowling Green State University, Bowling Green, USA; 2 Crystal Cove Conservancy, Newport Coast, USA; 3 icddr,b, Dhaka, Bangladesh; 4 Department of Anthropology and the Population Research Institute, The Pennsylvania State University, University Park, USA

**Keywords:** Fertility, Mortality, Quantum, Tempo, Hazard models

## Abstract

Mortality and fertility are important, intricately linked drivers of population change. Although past demographic research has focused largely on population-level mortality rates, individual-level mortality experiences can also shape fertility. The timing of mortality exposure over the life course might distinctly shape individual-level fertility behavior, with some time periods being more sensitive than others. Using detailed data from a sample of reproductive-aged women in Matlab, Bangladesh, this study investigates how mortality exposure, specifically to family bereavements, across three time periods (the focal woman’s childhood, adolescence, and post-marriage adulthood) is linked to fertility quantum and tempo. Results suggest exposure to kin mortality is associated with an increased fertility quantum despite delaying fertility tempo. We also find that mortality exposure post marriage (rather than in a woman’s childhood or adolescence) is the most crucial life course period in terms of the relationship between mortality exposure and fertility behavior. These findings distinctively underscore the need to account for mortality exposure in our understanding of individual-level fertility behavior, and also the importance of specific environmental components, such as the life course timing of mortality exposure, to better understand this relationship. These findings are consistent with previous literature on the association of mortality exposure with fertility, but our novel life course analyses contradict expectations that early life mortality exposure will be especially influential on later fertility.

## Introduction

Mortality and fertility are important, intricately linked drivers of population change. Both historical demographic theories and recent demographic research posit that mortality can shape both the quantum (i.e., parity) and tempo (i.e., timing) of fertility (Kirk, 1996; Low et al., 2013; Mason, 1997), with much of this research pointing to a relationship between mortality exposure and higher quantum and faster tempo. Nevertheless, there remains a debate on the relevancy of mortality to fertility in modern post-demographic transition societies where mortality rates are often lower and stabler than the past and fertility is often below or close to replacement level.

Although most research has focused on the connection between population-level mortality rates and fertility outcomes, the more limited research focused on individual-level mortality exposure has also shown a relationship with individual-level fertility (Pepper & Nettle, 2013; Rodgers et al., 2005; Sandberg, 2006; Zhang & Zhang, 2005). Research suggests that individual exposure to bereavements (Pepper & Nettle, 2013; Sandberg, 2005, 2006; Smith-Greenaway et al., 2022), deaths of key family members (Okun & Stecklov, 2021), exposure to homicide (Weitzman et al., 2021), or thoughts of mortality (Zhang & Zhang, 2005; Zhou et al., 2008) can lead to preferences for high fertility, reduced exertion of control over fertility, and earlier preferred and/or realized ages at first birth. Indeed, there is reason to suspect that these individual-level, and thus more direct, forms of mortality exposure may be more important, and/or more precise, predictors of fertility (Nobles et al., 2015; Pepper & Nettle, 2013; Rodgers et al., 2005; Sandberg, 2006; Zhang & Zhang, 2005) than regional or country-level mortality rates.

Moreover, some research suggests that the timing of mortality exposure over the life course may have a distinct relationship with fertility, with some time periods being more sensitive than others (Coall et al., 2016; Pepper & Nettle, 2013; Shenk & Scelza, 2012). Although research has documented that mortality exposure during different periods of the life course is associated with fertility (Chisholm et al., 1993; Okun & Stecklov, 2021; Pepper & Nettle, 2013; Shenk & Scelza, 2012; Weitzman et al., 2021), past research has often looked at only single periods of the life course instead of comparing mortality exposure across periods. The limits of prior investigations mean that we do not have a clear understanding of when in the life course mortality exposure is most consequential for fertility behavior. It is possible that mortality exposure might be highly influential on fertility at certain points in the life course while exposure in other time periods may have little impact.

Additionally, when the association between mortality exposure and fertility are considered, a common focus is on experiences of infant and child mortality (Hossain et al., 2007; Kirk, 1996; Mason, 1997; Sandberg, 2006). One well-known relationship is that exposure to infant and child mortality often encourages higher fertility quantum and faster tempo due to replacement or insurance strategies so that women will achieve their parity goals (Kirk, 1996; Sandberg, 2006). However, recent research finds that all-age mortality, and even simply adult mortality, can also shape fertility quantum and tempo (Nobles et al., 2015; Okun & Stecklov, 2021; Smith-Greenaway et al., 2022; Weitzman et al., 2021). Comparative work on the influence of the age of the deceased has also not been done. A comparison of how the age of the deceased shapes fertility quantum and tempo is needed to better understand whether the relationship between mortality and fertility extends beyond infant and child deaths, and, if so, which age groups do and do not have effects. Older adult deaths, for example, are more expected than child and infant deaths which might factor in, and potentially mitigate, their influence on family planning decisions.

In this study, we leverage unique and detailed individual-level mortality and fertility data from a sample of reproductive aged women in Bangladesh to fill three critical gaps in the demographic literature. First, we explore how individual-level mortality exposure, measured by the number of kin deaths, shapes fertility behavior. Past research has demonstrated that familial deaths (Beaujouan & Solaz, 2022; Okun & Stecklov, 2021; Shenk & Scelza, 2012) are associated with subsequent fertility behavior.^1^ Second, we compare how mortality exposure to kin deaths in the focal women’s childhood, adolescence, and post-marriage adulthood is linked to fertility quantum and tempo. Lastly, we evaluate whether the age of decedents matters, comparing the kin deaths of adolescents and reproductive aged adults against the deaths of post-reproductive adults to test their associations with fertility.

## Background

### Study Setting

Our study setting is Matlab, Bangladesh, a rural area in Chandpur District. Although agriculture is the dominant economic system in this area, it has steadily declined as a primary occupation in favor of market-engaged occupations in the past few decades as land ownership has decreased and access to other labor markets has increased (Alam et al., 2017; Kabeer, 2001). Although this region is known for a rapid and dramatic fertility transition from the 1960s through 1990s (Shenk et al., 2013; ICDDR, 2021), in the last two decades Matlab has retained a relatively steady post-transition fertility rate above replacement level with a current total fertility rate (TFR) of 2.6 (ICDDR 2021). A component of this above-replacement birth rate is early entry into marriage and early ages at first birth (a third of women are married under age 18) (ICDDR, 2021). It is common for women in Matlab to have their first child early, within the first year or so of marriage, followed by a relatively long birth interval before a second child is born, and in some cases another long interval before a third. Contraceptive access and use is high in Matlab, allowing for efficient control over the timing of births and the cessation of reproduction. Although infant and child mortality was once high in this region (e.g., an infant mortality rate (IMR) of nearly 200 during diarrheal epidemics in the 1960s and 1970s), these rates have decreased in the past several decades to a current IMR of around 25, making the mortality environment much more stable than in the past (ICDDR, 2021).

### Mortality Exposure Over the Life Course and Fertility

Life course theory emphasizes that the age of exposure to a stressor matters for its relationship to later life attitudes, behavior, and other outcomes (Elder, 1998; Torche, 2019). Risk of exposure to stressors varies with age (Hill, 1993; Sear et al., 2016), and sensitivity to stressors changes over the life course: an experience during a more sensitive period will shape a trait more than the same experience during a less sensitive period (Fawcett & Frankenhuis, 2015). Research supports that the relationship between mortality exposure, particularly the bereavement of a close friend or family member, and fertility differs based on the age of exposure. However, most studies either (a) compare early childhood or adolescence against current/adult exposure rather than comparing across multiple time periods (e.g., Chisholm et al., 1993; Coall et al., 2016; Quinlan, 2010; Shenk & Scelza, 2012), or (b) compare across populations with different local mortality rates rather than comparing individual differences in exposure within populations (e.g., Kirk, 1996; Low et al., 2013; Mason, 1997). Thus, it is still uncertain how the timing of mortality exposure over the life course shapes fertility and whether there is any particular stage in the life course in which mortality might be the most impactful.

#### Mortality Exposure in Childhood and Adolescence and Fertility

High local mortality rates in early childhood, as well as adolescence, are associated with an early age at sexual debut and first birth, as well as increased sexual risk taking and higher fertility (Beaujouan & Solaz, 2022; Chisholm et al., 1993; Coall et al., 2016; Pepper & Nettle, 2013; Placek & Quinlan, 2012; Simpson et al., 2012). Much of the research that assesses the relationship between childhood or adolescent mortality exposure and fertility approaches the topic through a developmental framework (Chisholm et al., 1993; Placek & Quinlan, 2012; Quinlan, 2010), predicting that mortality exposure in childhood and adolescence will mold individual responses to later environmental cues and thus fertility behavior.

The few studies that have evaluated whether childhood or adolescent mortality exposure has the greater influence over subsequent fertility largely show that childhood exposure to close bereavements outweighs adolescent exposure in studies set in rural Dominica (Quinlan, 2010), the United States (Simpson et al., 2012), and in crossnational research (Placek & Quinlan, 2012). However, mortality exposure in adolescence may moderate the association with exposure in childhood depending on the amount of childhood exposure (Placek & Quinlan, 2012; Quinlan, 2010). For example, when a woman had high exposure to mortality in childhood there was little evidence of a relationship between later mortality exposure and fertility tempo, yet when she had low exposure to mortality in childhood, exposure in adolescence shaped reproduction (Quinlan, 2010). Other work, from Bangalore, India, a relatively similar setting to Matlab, Bangladesh, suggests a stronger link between mortality exposure in adolescence and fertility, compared to mortality exposure in childhood, at least when considering paternal death (Shenk & Scelza, 2012).

#### Mortality Exposure in Adulthood and Fertility

Yet women are more likely to experience and be aware of mortality in adulthood as their social networks become larger, including through marriage, and older relatives continue to age and encounter higher mortality risks (Sandberg, 2005, 2006). Moreover, in adulthood, individuals become more aware of society in general including relevant social trends, for example, research has positively linked mortality and fertility through a shared need to rebuild communities among adults (Nobles et al., 2015; Rodgers et al., 2005). Thus, it is possible that this greater awareness of or exposure to mortality may mean that adulthood is the most influential period in shaping fertility. In Matlab, Bangladesh, for example, it is common for women’s networks to expand through marriage as they typically move to live with their husband’s kin—effectively doubling their network of kin and neighbors. Similar to research set in a small village in Nepal (Sandberg, 2005, 2006), Bangladeshi women are aware of the mortality that occurs in their adult networks. This awareness of mortality is a likely mechanism by which mortality exposure may shape adult fertility behavior.

In adulthood, individuals are likely more concerned with their own mortality and thus might also be more sensitive to experiences of grief than in childhood or adolescence. Past research has found a positive connection between mortality and fertility due to how it may reshape individuals’ perceived mortality risks (Low et al., 2013; Zhang & Zhang, 2005), suggesting that exposure to mortality may speed up or increase individuals’ fertility if they perceive their own life expectancy to be shortened. For example, epidemics such as HIV/AIDs have been shown to shape perceptions of own life expectancy which might in turn influence fertility (Low et al., 2013; Trinitapoli & Yeatman, 2011). Fertility might also be delayed in adulthood due to grief (Okun & Stecklov, 2021) or via stress-induced miscarriages (Torche, 2011). Mortality exposure in adulthood may also shape fertility through its influence on financial and childcare resources resulting from familial deaths (Grundy & Henretta, 2006; Harknett, et al., 2014; Okun & Stecklov, 2021). The sum of this past research highlights the necessity of investigating multiple life periods simultaneously to fully comprehend the relationship between the age at mortality exposure and fertility behavior.

### Age of the Deceased

Most research on how the age of the deceased relates to fertility has focused on the relationship between infant and child mortality and fertility (Hossain et al., 2007; Sandberg, 2006). However, recent research finds that adolescent and adult mortality can also shape fertility (Nobles et al., 2015; Okun & Stecklov, 2021; Weitzman et al., 2021). Although less explored, deaths of post-reproductive aged adults may also influence fertility decision-making. The death of a dependent older relative may free up a reproductive-age individual’s financial resources or caregiving time, thus increasing fertility quantum and/or tempo (Grundy & Henretta, 2006; Harknett et al., 2014). Similarly, the receipt of an inheritance could increase fertility behavior (Hansen & Wiborg, 2019; Korom, 2018). In contrast, the death of a more independent older relative might reduce fertility or delay births due to a reduction in childcare support (Okun & Stecklov, 2021). This could be particularly important in a context such as Bangladesh where there is a high volume of instrumental social support for childrearing (Shenk et al., 2021). In sum, research finds that mortality of all ages can shape fertility quantum and tempo (Okun & Stecklov, 2021; Pepper & Nettle, 2013).

We expect that particularly in a post-demographic transition society, such as Bangladesh, with low fertility and mortality rates, and relatively low rates of infant and child mortality, that fertility behavior will be shaped by mortality regardless of the age of the deceased, although the processes through which it operates may vary. However, past research provides an uncertain prediction on what time in the life course mortality exposure is most consequential for fertility. One perspective is that early life exposure might be the most critical for fertility behavior as it influences the mindset of individuals and thus the life course of individuals relevant to family formation and fertility planning. Conversely, an alternative perspective might suggest that mortality exposure in adulthood is the most consequential as fertility decisions are actively being made during this time.

## Methods

### Data Description

Respondents were randomly drawn from a complete list of ever married women aged 19–33 who lived in the Matlab Health and Demographic Surveillance System (HDSS) area in 2019 (Alam et al., 2017), a demographic surveillance site in operation since the late 1960s. Married women were selected as nonmarital births were, and still are, very rare in this region. The age range was chosen to focus on women who are actively engaged in fertility decision-making. The women in our sample play a large role in their reproductive decisions with over 95% identifying that they have “a lot” of influence or “total control” over how many children they have.

Our sample comprises 301 women. Although this sample size is modest in comparison to larger demographic datasets such as the Demographic and Health Surveys (DHS), past demographic studies have leveraged unique mortality and fertility data with comparable sample sizes to provide novel insights when relevant data do not exist in larger datasets (Sandberg, 2005, 2006; Weitzman et al., 2021). Duncan (2008) argues that smaller samples can sometimes be particularly effective when hypothesis testing rather than focusing on population composition. Additionally, power analyses were computed estimating a 5%-level two-sided test that concluded the current sample size has excellent power to detect significant differences.

### Measures

#### Fertility Measures

To assess fertility behavior, we examine the number of children ever born and having a first birth as dependent variables. These measures encompass dimensions of both fertility quantum (i.e., the number of births) and tempo (i.e., the timing of births). See Appendix A for the survey questions these measures are from.

#### Mortality Measures

Our key independent variables are self-reported mortality exposures– recalled familial deaths across the life-course. We do not investigate deaths of women’s own children due to the low infant mortality rate in the area. Our data provide estimates of mortality exposure during three distinct time periods: childhood (birth to age 11), adolescence (age 11 to marriage), and post marriage, which we consider adulthood. Due to the early ages of first marriage in this area, we recognize that post marriage might include individuals who are either adolescents or adults. However, we investigate these time periods distinctly, as strong social norms discourage premarital sex and childbearing.

For each period, women were asked to report on both sibling and parental deaths. They were also asked “How many of your close friends or close relatives died, not including siblings or parents?” Respondents were then asked to list, in roster form, details about each death experienced in each period of the life course. In the roster, however, respondents only identified deaths among familial relations, a fact that is not surprising given that kin (both natal and marital) comprise a very large proportion of women’s social networks in this region (Lynch et al., 2022). Our analysis thus focuses only on kin deaths.

These data allow us to assess the counts of mortality exposure in two ways: as a dichotomous measure (experienced no deaths as zero and experienced any deaths as one) and as a continuous measure of total deaths experienced. To ensure proper causal ordering, we adjust all mortality measures by the timing of an individual’s own fertility. For example, if a woman has her first birth at 20, we remove all deaths experienced after this birth in analyses where first birth is the outcome variable.

Our data are retrospective and thus may be subject to recall bias, yet recall biases in mortality (and similar variables) are most prevalent among older age groups (Gu & Dupre, 2008; Van de Mheen, et al., 1998) and more likely to occur for infant than adult deaths (Roberts et al., 2004). Women in our sample are relatively young (19–33) and thus are more likely to accurately recall mortality than if our sample were from an older age group. Likewise, the survey requested data on deaths of all ages rather than just infant deaths and we expect this reduces recall bias.

Our mortality data has a larger concentration of deaths in focal individuals’ post-marriage period in comparison to other times in the life course. Better recall of recent deaths might contribute to this higher frequency of deaths reported post marriage than in other periods. However, increases in women’s social network sizes post marriage to include in-laws, broader family and friends, and new neighbors (patrilocal social norms mean that women generally move to their husband’s location at marriage), and the aging of members of their networks, including both parents and in-laws, are likely to be important factors. Our mortality data are thus consistent with a life course explanation for the increase in mortality exposure post marriage, as many of the reported deaths include older relatives, including in-laws.

#### Sociodemographic Measures

We control for multiple sociodemographic variables, including age, education, and relative wealth. Women’s age is based on date of birth from HDSS data or National ID Cards. Education is measured as years of schooling completed. We assess relative wealth using a subjective measure inspired by the MacArthur Ladder (Adler & Stewart, 2007) that we have modified for local circumstances. The question asks, “Please think about others around you now. Think about how well off they are, and whether they eat well, get new clothes and shoes when they want them, and have money to buy nice things for their homes. How wealthy is your family compared to other families?” This measure asks respondents to consider the past 12 months and answer either “less,” “average,” or “more.” As a robustness check, we also assessed how this measure compared to other measures of wealth, such as monthly income, and whether our results were sensitive to a particular measure of wealth; we found this measure to be comparable to those.

### Analytical Strategy

First, we describe our data using descriptive statistics from our analytical sample. These highlight the averages and percentages of fertility, mortality, and sociodemographic variables. These also show variation in mortality exposure by timing in the focal individual’s life course, presented as a binary measure of any mortality exposure and a continuous variable that captures the magnitude of exposure.

For our main results, we analyze our measures of fertility behavior (i.e., children ever born and first birth) using Cox Proportional Hazard Models or Ordinary Least Squares regression models. We use Cox Proportional Hazard Models for the analysis of the hazard of experiencing a first birth. In our sample, 283 women have had their first birth (18 women are treated as right censored). Due to this context’s sustained birth rate above replacement level fertility, and strong social norms that most women should marry and that all married women should have children, it is expected that virtually all women will have at least one child, and most women will have two or more children. Given that births rarely occur outside of marriage, the hazard of first birth begins at the age of first marriage. Cox Proportional Hazard Models allow us to retain our full sample, compare the hazards of women with and without mortality exposure, and explore how differences in exposure shape the timing of births (Allison, 2014).^2^ When we assess the number of children ever born as an outcome, we use ordinary least squares regression.^3^

First, we estimate models for the overall number of deaths experienced and their relationship with fertility behavior. Next, we use our mortality measures as binary variables to assess the relationship between any mortality exposure and fertility behavior by life course timing. Last, to understand if magnitude of exposure across the life course is associated with variation in the relationship between mortality exposure and fertility, we investigate the association between the number of deaths experienced post marriage and fertility. In all models, we control for age, education, and relative wealth.

We also assess whether the association between mortality and fertility is responsive to the age of the deceased. We classify post-reproduction deaths as 55 years and older and working age deaths as 15 to 54 years old as these classifications are consistent with local mortality patterns in which mortality risks rise notably after age 55. Additionally, in this context it is common for people to start work at 15 or enter marriage in their later teenage years, which means the consideration of adulthood is different in comparison to other contexts.

## Results

### Descriptive Statistics

The top of Table 1 shows the descriptive statistics for our mortality measures. It is common in our sample for women to experience the death of a close friend or relative at some point during the life course (89%). Women, on average, had experienced 2.4 close deaths at the time of the interview, with a mean of 1.3 deaths post marriage. We also see that mortality exposure slowly increases across the life course with the lowest exposure in childhood (33% of women), followed by adolescence (44%), and then the highest by the post marriage period (67%). Our sample of women has a mean of 1.6 births. The average age of first birth is 20.4.

Our sample comprises women who are in their childbearing years (women’s average age was 26.2 years at time of interview). Most women have less than 10 years of total education and perceive their household wealth to be average compared to other families (68.8%), though some consider their wealth to be less than others (17.9%) and a few perceive their household wealth to be greater than others (13.2%).

### Fertility Behavior

Table 2 presents the results of our models that examine the association between mortality exposure and the number of children ever born. Model 1 shows that across the life course, the total number of deaths focal individuals are exposed to is not associated with the number of children ever born. However, it may be that parity is sensitive to the timing of mortality exposure. Model 2 suggests that mortality exposure post marriage is the most crucial period in terms of consequences for number of children ever born. We find no significant associations between deaths experienced in childhood or adolescence and the number of children ever born. However, results suggest that mortality exposure post marriage is associated with an increase in fertility quantum. Experiencing at least one close death post marriage is associated with a 0.20 increase in the number of children ever born (CI: 0.04, 0.36). This result implies that exposure to mortality post marriage may increase parity. Next, we assess whether our results are sensitive to the magnitude of mortality exposure (i.e., the number of deaths experienced). Model 3 (total deaths post marriage) shows the results of these models when mortality is treated as a continuous instead of a binary variable. Each additional death experienced is associated with an 0.08 increase in parity (CI: 0.01, 0.14). Overall, these results are consistent with the binary results presented in Model 2.

Table 3 presents similar results as Table 2 but focuses on the hazard of a first birth. Model 1 shows that across the life course, the total number of deaths women are exposed to is negatively associated with the hazard of a first birth (HR: 0.90, CI: 0.83, 0.98). In Model 2, experiencing at least one family death post marriage is associated with a 34% (100–66%) decrease in the hazard of experiencing a first birth (C.I.: 0.50, 0.87). This result suggests that exposure to mortality may delay women’s reproduction by increasing the age at which a woman has her first birth. In Model 3, we see a similar association when we look at the magnitude of exposure to death post marriage. Each additional death experienced post marriage is associated with a decrease in the hazard of a first birth (HR: 0.70, CI: 0.57, 0.87).

### Age of the Deceased

Tables 4 and 5 present results examining exposure to deaths among working age (aged 15–54) and post-reproductive individuals (aged 55 +). Results indicate consistent associations of fertility with exposure to both types of deaths: Fertility increases in quantum but decreases in tempo with mortality exposure, particularly post marriage. Exposure to working age deaths post marriage is associated with a 0.21 increase in children ever born (CI: 0.02, 0.40) and a 42% (100–58%) decrease in the hazard of experiencing a first birth (CI: 0.42, 0.81).

Comparable results are found for exposure to deaths of post-reproductive individuals. Exposure to at least one death post marriage is associated with a 0.21 increase in children ever born (CI: 0.05, 0.37). Results also indicate that exposure to at least one death of a post reproductive person is associated with a 47% (100–53%) decrease in the hazard of experiencing a first birth (CI: 0.36, 0.78).

## Discussion

Our study examined the relationship between mortality exposure and fertility quantum and tempo using unique and detailed survey data from a sample of women in Matlab, Bangladesh, with the goal of providing insight on life course processes with implications for understudied aspects of the individual-level relationship between fertility and mortality. We sought to address the open questions of how individual exposure to kin mortality (bereavements) overall, as well as exposure at certain life course periods, are related to fertility by focusing on the timing of mortality exposure in childhood, adolescence, and post-marriage adulthood. Additionally, as past research has shown fertility might respond distinctly to the mortality of people of different ages, we use our detailed mortality data to examine whether the age of the deceased shapes the relationship between mortality and fertility.

With respect to mortality exposure, our findings indicate support for previous research that suggests individual exposure influences fertility quantum and tempo (Nobles et al., 2015; Pepper & Nettle, 2013; Rodgers et al., 2005; Sandberg, 2006; Zhang & Zhang, 2005). Our results imply that exposure to bereavements has a positive relationship with fertility quantum, as we find a consistent positive association between mortality exposure and the number of children ever born. Our results also suggest that exposure to kin deaths is associated with a slower fertility tempo. Thus, women who are exposed to kin mortality may have their fertility tempo temporarily disrupted but still have high parities.

In terms of when in the life course kin mortality is most related to fertility, we find that post marriage is the most crucial period. These results highlight that mortality exposure might be most impactful on fertility behavior when women are actively having children. Contrary to some other findings in the literature noted above (e.g., Chisholm et al., 1993; Coall et al., 2016; Quinlan, 2010), these results suggest that fertility behavior may be more strongly related to women’s current than past environment. Currently we cannot be sure if our finding differs from those of others because our data are better at tracking timing of mortality exposure or whether this relationship is different in different environments, but this remains a fruitful area for future exploration. Lastly, our results suggest that deaths of both younger (working age) and older (post reproduction) adult kin are associated with increased fertility quantum and a slower tempo.

Regarding the timing of fertility, our results suggest that mortality exposure post marriage can delay first births. These results concur with past research finding that bereavements of close family relations can stall fertility timing (Okun & Stecklov, 2021). These delays may be related to multiple factors including a lack of economic and/or support resources post-mortality (Okun & Stecklov, 2021) or grief and stress-related factors (Torche, 2011). Research showing a faster fertility tempo response to other kinds of mortality exposure such as low life expectancy (Low et al., 2013), funeral attendance (Smith-Greenaway et al., 2022), or own child death (Kirk, 1996) suggests that such effects can occur through different processes, underlining the potential for multiple pathways in the relationship between mortality exposure and fertility tempo. Specifically, while exposure to high levels of general mortality may motivate an increased childbearing tempo (Low et al., 2013; Smith-Greenaway et al., 2022), and own-child deaths are well-known to motivate replacement fertility to achieve parity goals, our work along with that of others (Okun & Stecklov, 2021) suggests that the disruption caused by the death of an adult family member may temporarily delay fertility due to psychological and economic stressors. In the context of rural Bangladesh, where social support from kin is very important in childbearing, it is highly plausible that the loss of a key family member might disrupt the timing of a first birth. This could be due to economic hardship related to loss of income or the need to pay for medical bills, loss of help with childcare and housework, stress and depression after the death of a close relative, the need for travel to manage family affairs, and/or an increased likelihood of family relocation.

However, these stalls in tempo may not decrease overall fertility, but in fact may even do the opposite, as our results also indicate a positive relationship between mortality exposure post marriage and the number of children ever born. This positive relationship aligns with past research that frequently finds a positive relationship between mortality exposure and fertility (Nobles et al., 2015; Pepper & Nettle, 2013; Rodgers et al., 2005). It is also consistent with fertility patterns in this context as women start childbearing early and only have two to three children, meaning that temporary stalls in tempo can easily be recouped in terms of quantum over time. It appears that although higher levels of general mortality exposure, in line with theory and many empirical findings, work to motivate higher fertility at a faster pace, the challenges of dealing with family deaths may slow childbearing in the short term even while encouraging higher fertility in the longer term. It is possible these increases in parity occur in our sample of Bangladeshi women due to multiple mechanisms. Women and families may feel the need to replace family members and community following mortality exposure, which has been shown in similar contexts such as Indonesia (Nobles et al., 2015). They may also view the world as a riskier place and be motivated to have more children as a hedge against such mortality risks, an argument that is expected to apply more strongly in the context of low and middle income countries where mortality risks are harder for individuals to control (Placek & Quinlan, 2012; Quinlan, 2007).

These results also build on the understanding of mortality exposure over the life course and how it can contribute to fertility. Although past research tends to focus on childhood and adolescence due to developmental theories of the importance of sensitive periods that might shape later life behavior (Chisholm et al., 1993; Placek & Quinlan, 2012; Quinlan, 2010), our study suggests that exposure to kin mortality in post-marriage adulthood is more clearly related to fertility. This finding might occur because as women age, their environment and risk of mortality exposure fluctuates (Hill, 1993; Sear et al., 2016). Additionally, exposure post marriage might be most related to fertility as individuals may have a higher awareness of mortality than when they were younger, better enabling a link with both their own perceived life expectancy and their perceptions of infant and child mortality risk (Low et al., 2013; Zhang & Zhang, 2005). Additionally, exposure to kin mortality might shape fertility post marriage through important pathways such as availability of financial resources or caregiving (Grundy & Henretta, 2006; Harknett et al., 2014; Okun & Stecklov, 2021). Finally, mortality exposure in post-marriage adulthood might be a more accurate cue to the prevailing mortality conditions, making women’s attention to this factor strategic in terms of the best decisions regarding fertility and investment in children. These factors are all likely contributors to the finding that mortality exposure in post-marriage adulthood is more related to fertility than mortality exposure at earlier points in the life course.

Interestingly, our results also highlight that the modern relationship between mortality and fertility may not be tied to the age of the deceased, at least not for the deaths of adults. Past research has found that fertility is responsive to mortality from adult deaths (Okun & Stecklov, 2021; Pepper & Nettle, 2013) and all aged community deaths (Nobles et al., 2015; Weitzman et al., 2021). Our results concur with past research as we find similar results for the association of both working age and post-reproductive adult deaths with fertility behavior. These types of deaths, regardless of age range, show similar positive effects on quantum, stalling effects on the tempo of births, and are most influential if mortality exposure occurs in post-marriage adulthood.

Our results highlight how informative individual-level mortality data are for understanding fertility far beyond the traditional idea that infant mortality may result in replacement births (Kirk, 1996; Mason, 1997). We find that exposure to the deaths of adult kin is especially related to fertility, suggesting that the relationship between these two demographic processes has been understudied in demography. Our results also suggest the importance of investigating both quantum and tempo to better understand the implications of mortality exposure on fertility. Future research should continue to study these measures collectively to further build on demographic insights, as the interplay between the two is key to understanding both individual- and population-level fertility. Additionally, future research must continue to investigate the mechanisms by which fertility might be disrupted by mortality, including examining the different pathways that may exist between general mortality exposure and exposure to the deaths of kin.

Although our study leverages unique and detailed mortality and fertility data from Bangladesh, it is not without limitations. Although detailed, our sample size is small in comparison to commonly used demographic datasets such as the DHS, yet these rarely contain the level of detail regarding mortality exposure needed for such analyses. This limited sample size does not allow us to examine whether recent deaths, such as in the past year, or those from any particular point post marriage, have more influence on fertility behavior. Yet we have substantial power for our analyses, and our key findings are robust, underlining our previous point that even small samples are useful if they contain unique data. Although the women in our sample are young, which helps mitigate recall bias, it is also possible that recent deaths (i.e., post marriage) are still more likely to be recalled than deaths in childhood and adolescence, driving the significance of post-marriage deaths versus other time periods. However, because we dichotomize mortality exposure in most of our results, we can be more confident in their robustness. Future research should continue to explore lifetime mortality exposure and how certain life course periods may uniquely shape fertility behavior. In our analysis, we only use data from women, which means we do not directly capture their husbands’ mortality experiences. Yet, given that women’s family networks virtually always include their in-laws, and in-law deaths are very common in our data, it is likely that we still capture the experiences of key deaths in the husband’s family and social network in our analysis. Future research might consider including data from men’s perspectives to better understand the important role they often play in fertility decisions.

This study builds on past research by furthering a demographic understanding of the connection between mortality and fertility. We find a clear relationship between kin mortality exposure and higher fertility quantum yet slower tempo. We also find evidence that mortality exposure in post-marriage adulthood, rather than at earlier ages, drives the association between mortality exposure and fertility—and this relationship does not appear to vary across the age of the deceased. These findings distinctively underscore the need to account for mortality exposure in our understanding of fertility, and the importance of specific environmental components, such as the life course timing of mortality exposure, to better understand this relationship. More broadly, however, this study highlights the need for comprehensive research on the connection between mortality and fertility—and how this might vary across types and timing of mortality exposure. We anticipate that some aspects of these results will be potentially generalizable to any setting which shares similar social and ecological factors, while other aspects are likely very widely generalizable given their strong fit with the theoretical literature.

Further research is needed before we know enough to implement sound policy, yet this work does have some policy implications. While demographers and population researchers are aware that uncertainty can affect demographic decisions, apart from infant mortality the effects of mortality risk on fertility have been understudied. Our work, along with that of a few others (Nolin & Ziker, 2016; Smith-Greenaway et al., 2022; Trinitapoli & Yeatman, 2011), highlights the importance of a more comprehensive understanding of the implications of risk and uncertainty—including individual levels of mortality exposure and the timing of that exposure—for fertility, and suggests that policymakers may need to consider a broader set of circumstances in understanding the conditions under which fertility may rise or fall. A second implication of our work is our finding that mortality exposure simultaneously increases fertility quantum while slowing tempo. These countervailing directional effects, while not unprecedented (Nobles et al., 2015; Okun & Stecklov, 2021), suggests a need for careful study of the complex patterns of fertility under conditions of risk and uncertainty. It also suggests a need for careful consideration of fertility policy, as delayed first birth is often treated as a straightforward proxy of fertility decline, yet in some circumstances this may not be accurate or appropriate.

## Figures and Tables

**Table 1:** Sample descriptive statistics

Variables	Mean/Percent	Stand. Dev.	Min.	Max.	Obs.
*Mortality (Binary Measure)*					
Exposed to Any Deaths	89%		0	1	310
Exposed to Any Deaths in Childhood	33%		0	1	310
Exposed to Any Deaths in Adolescence	44%		0	1	310
Exposed to Any Deaths Post Marriage	67%		0	1	310
*Mortality (Continuous Measure)*					
Number of Deaths	2.37	1.86	0	9	310
Number of Deaths in Childhood	0.53	0.96	0	7	310
Number of Deaths in Adolescence	0.61	0.83	0	4	310
Number of Deaths in Adulthood	1.23	1.25	0	6	310
*Fertility*					
Number of Children Ever Born	1.62	0.83	0	5	310
Age at First Birth	20.42	2.52	14	29	291
*Sociodemographic*					
Age	26.19	3.69	19	33	310
Education	8.77	2.74	0	14	310
Relative Wealth					
Less	17.42%				54
Average	69.35%				215
More	13.23%				41

Notes: Age at first birth descriptive statistics exclude censored values. With censored values the mean is 20.6 and standard deviation is 2.6

**Table 2:** Estimates for the relationship between mortality exposure and children ever born

	Children ever born		
	Model 1	Model 2	Model 3
*Number of Deaths*	.01 (−.03, .05)		
*Exposed to Any Deaths in Childhood*		−.01 (−.18, .15)	
*Exposed to Any Deaths in Adolescence*		−.11 (−.27, .05)	
*Exposure to Deaths Post Marriage*		.20* (.04, .36)	.08* (.01, .14)
*Age*	.12*** (.10, .14)	.12*** (.10, .14)	.12*** (.10, .14)
*Education*	−.04* (−.06, −.01)	−.03* (−.06, −.00)	−.03* (−.06, −.01)
*Relative Wealth (Ref.: Less)*			
Average	−.07 (−.27, .14)	−.06 (−.27, .15)	−.06 (−.26, .15)
More	−.20 (−.49, .09)	−.22 (−.50, .07)	−.19 (−.47, .10)
*Constant*	−1.24*** (−1.91, −.57)	−1.29*** (−1.96, −.62)	−1.25*** (−1.91, −.59)
	N = 301		

*p*Coefficients with 95% confidence intervals in parentheses

*** *p* < 0.001,

** *p* < 0.01,

* *p* < 0.05,

+ *p* < 0.10

**Table 3:** Estimates for the relationship between mortality exposure and first birth

	First birth		
	Model 1	Model 2	Model 3
*Number of Deaths*	.90* (.83, .98)		
*Exposed to Any Deaths in Childhood*		1.04 (.80, 1.34)	
*Exposed to Any Deaths in Adolescence*		.86 (.68, 1.10)	
*Exposure to Deaths Post Marriage*		.66** (.50, .87)	.70** (.57, .87)
*Age*	.93*** (.90, .96)	.93*** (.89, .96)	.93*** (.89, .96)
*Education*	.93** (.90, .98)	.93** (.89, .97)	.93** (.89, .97)
*Relative Wealth (Ref.: Less)*			
Average	.82 (.60, 1.12)	.81 (.59, 1.11)	.85 (.63, 1.16)
More	.66 + (.42, 1.01)	.69 (.45, 1.08)	.73 (.48, 1.13)
N = 301			

Estimates are presented as hazard ratios with 95% confidence intervals in parentheses

*** *p* < 0.001,

** *p* < 0.01,

* *p* < 0.05,

+ *p* < 0.10

**Table 4:** Results by age of the deceased for children ever born

	Deceased age 15–54	Deceased age 55+
*Exposed to Any Deaths in Childhood*	.02 (−.27, .31)	.01 (−.18, .19)
*Exposed to Any Deaths in Adolescence*	.02 (−.23, .28)	−.12 (−.29, .05)
*Exposed to Any Deaths Post Marriage*	.21* (.02, .40)	.21* (.05, .37)
*Age*	.12*** (.10, .14)	.12*** (.10, .14)
*Education*	−.04* (−.07, −.01)	−.03* (−.06, −.01)
*Relative Wealth (Ref.: Less)*		
Average	−.07 (−.27, .14)	−.05 (−.26, .15)
More	−.21 (−.49, .08)	−.17 (−.46, .11)
*Constant*	−1.15** (−1.82, −.49)	−1.14** (−1.80, −.48)
N	301	

Coefficients with standard errors in parentheses

*** *p* < 0.001,

** *p* < 0.01,

* *p* < 0.05,

+ *p* < 0.10

**Table 5:** Results by age of the deceased for first birth

	Deceased Age 15–54	Deceased age 55+
*Exposed to Any Deaths in Childhood*	1.10 (.71, 1.70)	.97 (.74, 1.29)
*Exposed to Any Deaths in Adolescence*	1.01 (.69, 1.49)	.84 (.64, 1.08)
*Exposed to Any Deaths Post Marriage*	.58** (.42, .81)	.53** (.36, .78)
*Age*	.92*** (.89, .96)	.93*** (.89, .96)
*Education*	.93*** (.89, .97)	.94** (.90, .98)
*Relative Wealth (Ref.: Less)*		
Average	.85 (.62, 1.16)	.87 (.64, 1.19)
More	.79 (.51, 1.24)	.69 + (.45, 1.07)
N	301	

Estimates are presented as hazard ratios with 95% confidence intervals in parentheses

*** *p* < 0.001,

** *p* < 0.01,

* *p* < 0.05,

+ *p* < 0.10

## Footnotes

1 Due to the low infant mortality rate in this context, we do not examine the deaths of women’s own children.

2 A common data problem when using Cox Proportional Hazard Models are ties in the data, which we use the Breslow method to manage (Allison, 2014; Singer & Willet, 2003).

3 When we limit the sample only to women who no longer intend to have children (n = 199), the results for the number of children ever born are similar to those presented with the full sample.
